# Immunological and virological questions for H5N1 pandemic emergence

**DOI:** 10.1093/immhor/vlaf062

**Published:** 2025-12-16

**Authors:** Mario A Peña-Hernández, Miyu Moriyama

**Affiliations:** Department of Immunobiology, Yale University School of Medicine, New Haven, CT, United States; Department of Microbial Pathogenesis, Yale University School of Medicine, New Haven, CT, United States; Department of Immunobiology, Yale University School of Medicine, New Haven, CT, United States

**Keywords:** avian influenza, cross-protective immunity, immune imprinting, pandemic potential

## Abstract

Zoonotic spillover of influenza A viruses into humans has repeatedly triggered pandemics throughout history. Since their emergence in the 1990s, H5N1 influenza viruses have significantly expanded their geographical range and host species, raising global concern about the potential for sustained human-to-human transmission. In this review, we examine the virological characteristics of currently circulating H5N1 strains, key molecular barriers limiting their spread among humans, and critical areas of future research to mitigate the ongoing H5N1 panzootic and prevent future pandemics.

## Introduction

Influenza A viruses have been a consistent pandemic threat.^1^ Throughout history, influenza A viruses have caused multiple pandemics with a wide variety of clinical respiratory manifestations that are primarily upper respiratory symptoms, but in some cases, they appear in the lower respiratory tract and as a severe pneumonia.^2^ Influenza A viruses of subtype H3N2 and H1N1 are currently considered human endemic and seasonal, albeit still causing millions of severe infections annually.^3^ While endemic influenza strains are now circulating in humans and the disease is commonly not severe, zoonotic strains with pandemic potential tend to be highly pathogenic to mammals, including humans.^4^ In particular, the highly pathogenic avian influenza virus (HPAI) subtype H5N1 has been described to cause severe lower respiratory symptoms that ultimately provoke pneumonia and respiratory failure with an estimated 30% to 50% fatality rate in humans.^3^ Since 2021, H5N1 influenza viruses have caused a dramatic increase in the number of outbreaks in migratory birds, wild mammals, and, more recently in the United States, cattle and poultry farms.^5^ This increase in circulating H5N1 infections in settings close to human contact underscores the urgent need to apply and improve measures to mitigate its spread. Frequent human encounters also increase the potential for H5N1 viruses to cause a human pandemic in the near future.

HPAI H5N1 was initially discovered in 1959 but later described as being capable of infecting humans in 1997 when a cluster of severely ill patients had atypical influenza infections in Hong Kong.^6^^,^^7^ Since then, sporadic cases of human infections have been identified in the past 2 decades, primarily among poultry workers and people who are in close contact with wild birds. Importantly, the emergence of a novel reassortant H5N1 from H5N8 strains (clade 2.3.4.4b) in 2021 has caused a substantial increase in outbreaks in migratory birds that has expanded the H5N1 variant into Asia, Europe, and North America.^1^^,^^5^

Influenza H5N1 viruses have evolved to interact with the avian α2,3-linked sialic acid receptors, which are widely expressed in the gastrointestinal tract of birds.^8^ In contrast, human influenza viruses primarily use α2,6-linked sialic acid receptors, which are abundantly expressed in the human respiratory tract.^9^ However, humans also express α2,3-linked sialic acid in their lower airway,^8^^,^^10^ making them susceptible to avian H5N1 viruses when exposed to high levels of viral infectious particles.^3^ The tropism of H5N1 viruses to the lower airway in humans is believed to be one of the main reasons for their high pathogenicity. Additionally, molecular features present in the hemagglutinin (HA) protein, such as a polybasic cleavage site to enhance processing of HA and viral entry, also contribute to its pathogenicity in mammals and birds.^11^ Importantly, due to the inefficient usage of human receptors and tropism for the lower airway, human-to-human transmission of H5N1 viruses has been described to be relatively inefficient. However, a constant circulation of avian influenza viruses in cattle and poultry might allow H5N1 viruses to adapt to human receptors.

**Figure 1.**
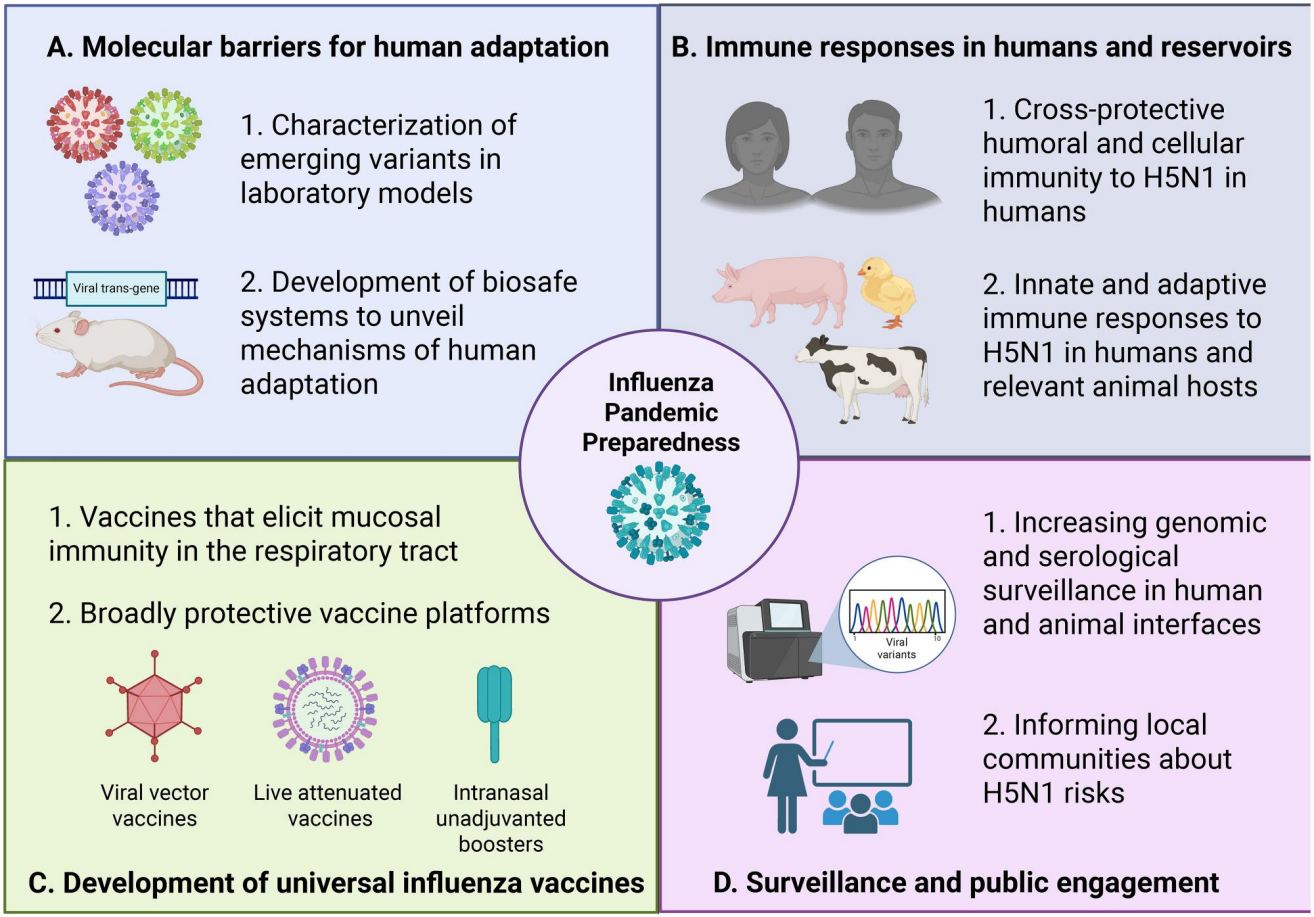
Research needed for potential avian H5N1 influenza pandemic preparedness. (A) Aspects to study from newly emerging avian H5N1 variants and mechanisms of human adaptation. (B) Importance of studying immune responses from human and animal reservoir infections. (C) Necessity to develop transmission-blocking vaccine strategies. (D) Importance of science communication to improve awareness and trust from the general public. This figure was created in BioRender (Peña-Hernández, M., 2025; https://BioRender.com/0f4usom).

Building on the other scientific reviews discussing historical, virological, and molecular insights of the recent emergence of HPAI H5N1^,1,3,12^ this manuscript will elaborate in the current knowledge of phenotypic assessment of circulating emerging H5N1 variants, how human immune responses could shape the pandemic potential of emerging influenza variants, and what research is critically needed to mitigate the spread of the current H5N1 panzootic and to prevent the emergence of the next influenza pandemic (Fig. 1).

**Figure:**
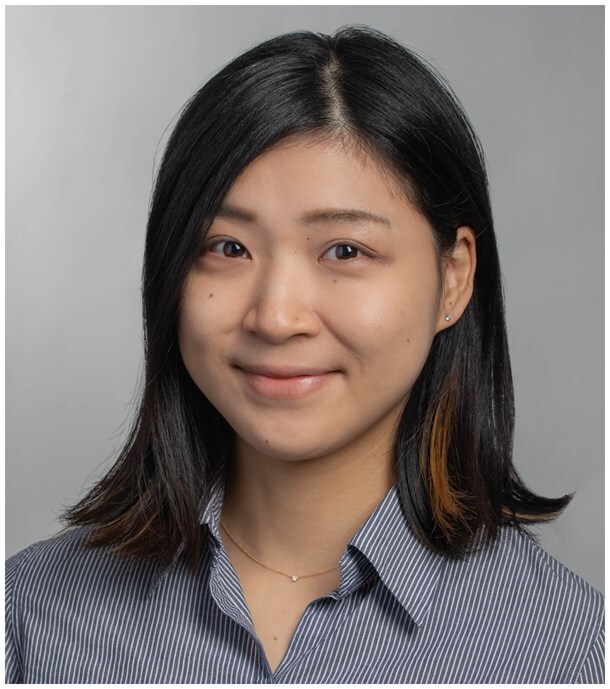
Miyu Moriyama, Ph.D., is an Associate Research Scientist in the Department of Immunobiology at Yale University. Her research focuses on how host, environmental, and viral factors shape innate and adaptive immune responses to respiratory viruses, with a particular emphasis on influenza. She has been recognized with multiple awards, including the Second Marie Sklodowska Curie Award from the Japan Science and Technology Agency.

## Recent host expansion of H5N1

The reassortment and emergence of H5N1 clade 2.3.4.4b since 2020–2021 have sparked global outbreaks in multiple host species. Migratory birds have likely seeded H5N1 outbreaks all across Asia, Europe, and America.^1^^,^^5^ In 2022–2023, fur farms in Spain and Finland (primarily minks) were affected by outbreaks. However, the situation was contained through the culling of animals in the affected farms.^13^^,^^14^ During late 2022 and 2023, South America suffered from multiple outbreaks of HPAI H5N1. Mass infections in marine mammals such as sea lions and sea birds (including penguins) were observed in Chile, Peru, Argentina, and other countries.^15–17^ Additionally, H5N1 viruses were also detected in the Antarctic region for the first time in 2023.^18^ Genetic sequencing of these viruses revealed the presence of mutations that have been described to provide mammalian adaptation. Thus, it is believed that multiple mammal-to-mammal transmission chains occurred during these outbreaks.^15^

In early 2024, H5N1 viruses were detected spreading among dairy farms in the United States.^19^^,^^20^ This was the first time that H5N1 was described to infect cattle. Since then, more than 1,000 dairy herds across the country have been affected by the H5N1 outbreaks.^21^ Viral phylogenetic analysis indicates that it was a single introduction from birds to cattle since all the affected herds possessed viral genomes belonging to the genotype B3.13 that clusters together.^22^ However, the genotype D1.1 was detected in cattle in early 2025 in the state of Nevada, indicating a second introduction of avian influenza viruses into cattle.^21^ While both genotypes B3.13 and D1.1 possess H5 from the lineage 2.3.4.4b, they possess non-HA viral segments that might influence a different degree of pathogenesis.^12^

In bovines, it has been observed that the H5N1 virus primarily replicates in the mammary glands,^19^^,^^23^ and it is speculated that transmission in dairy farms is mediated by contaminated equipment and devices used to obtain milk from the infected animals.^12^^,^^19^ Importantly, unpasteurized milk harbors a high infectious viral load of H5N1,^24^ and oral inoculation of raw milk from infected cattle has been shown to be fatal in mice.^25^ Additionally, H5N1 has been described to productively infect cats and mice near the affected dairy and poultry farms.^26^^,^^27^ This underscores the immediate risk for dairy workers exposed to infected herds and animals near these farms.

As of May 2025, an unprecedented number of 70 human cases have been identified in the United States since February 2024.^21^^,^^28^ The number of cases is likely underestimated due to the lack of specific H5N1 testing among dairy farms and other patients with respiratory symptoms. This highlights the need for increased mass testing for the detection of acute H5N1 infections, as well as serology approaches to uncover unidentified past infections in poultry and dairy workers (Fig. 1D). Interestingly, the case fatality rate of these H5N1 infections has not been as high as previously observed. There are a few potential reasons for this, including viral genetics and cross-protective immunity from human influenza virus strains. These points will be discussed in detail in the section of “Why does bovine-associated H5N1 exhibit attenuated pathogenesis in humans?”.

## Phenotypic assessment of emerging H5N1 variants

Some molecular features and mutations in avian influenza viruses to acquire efficient transmissibility in ferrets have been previously described, including specific mutations in HA that enable preferential binding to human receptors, PB2 (part of the polymerase complex), and neuraminidase (NA).^29–31^ However, other factors contribute to efficient transmission potential among avian influenza viruses, which will be elaborated in detail in the section “Molecular barriers for H5N1 transmission in mammalian species”. Additionally, it is also important to elucidate other determinants of influenza virus pandemic potential beyond receptor interactions. For instance, HA and virion stability to tolerate lower pH might impact the efficiency of establishing productive infections through the respiratory routes. To start elucidating these mechanisms, it is necessary to study how the emerging H5N1 variants behave in multiple laboratory animal models (Fig. 1A). Importantly, assessing how the emerging avian or cattle H5N1 variants behave in laboratory models may guide the risk stratification of these strains for pandemic preparedness.

In the laboratory setting, ferrets have been used to assess the transmission potential of influenza virus strains, as they express a similar sialic acid pattern across their respiratory tract as in humans. For instance, one of the first isolates of H5N1 2.3.4.4b obtained from a mink farm proved to be capable of transmitting in ferrets.^32^ A bovine HPAI H5N1 isolate was shown to cause lethal disease in mice and ferrets, albeit inefficiently transmitted among them.^33^ Mixed reports suggest that this isolate might or might not have the capacity to bind to human receptors.^33–35^ Moreover, experimentally infected cows confirmed their susceptibility and clinical manifestations, as well as reduction in milk productivity.^36^^,^^37^ In addition, macaques are susceptible to bovine H5N1 2.3.4.4b and cause mild and severe disease when inoculated intranasally and intratracheally, respectively.^38^

Human H5N1 2.3.4.4b isolates have also been characterized in ferret models. The human isolate from Texas (A/Texas/37/2024) has been shown to systemically spread and replicate, cause severe disease in ferrets and mice, and was transmitted among ferrets.^39^^,^^40^ In contrast, the human isolate from Michigan (A/Michigan/90/2024) exhibited limited replication out of respiratory tissues and moderate disease (nonlethal), yet remained capable of airborne transmission in ferrets.^41^ These notable differences in replication and pathogenicity may be linked to the presence of virulence-associated mutations in the Texas isolate, including E627K (change from glutamate [E] to lysine [K] at amino acid position 627) in PB2 and 142E in PA (subunit of the polymerase complex).

Pigs played a crucial role in the emergence of the 2009 influenza H1N1 pandemic.^42^ Regardless of avian or mammalian origin, all H5N1 2.3.4.4b isolates tested productively infected the lungs in a swine model. However, only mammalian isolates replicated in the upper airway demonstrated transmission capacity.^43^ Pigs are susceptible to a bovine 2.3.4.4b isolate and can transmit the virus in a direct contact setting.^44^ Since pigs are susceptible to both avian and human influenza viruses, the risk of potential reassortments between mammalian and avian strains is particularly higher in these animals.^45^ It is therefore important to study virology and immunology in pigs to better assess the risk of possible reassortment with human seasonal or swine strains (Fig. 1B). Additionally, increasing surveillance in pig farms, as well as in other susceptible animal hosts and their ecosystems, would enable effective risk stratification at the wildlife–livestock–human interface in alignment with the One Health approach.^46^ This approach has been successfully implemented in dairy farms in California, showing the presence of H5N1 infectious particles in the exhaled breath of cows, air and water streams, and milk from the farms.^47^

## Molecular barriers for H5N1 transmission in mammalian species

Identifying the molecular barriers preventing the avian influenza viruses from efficient human-to-human transmission is key to assessing the pandemic risk of H5N1 and other avian influenza viruses. To efficiently spread among humans, avian influenza viruses must adapt to overcome multiple layers of molecular barriers.^48^ This includes receptor compatibility and efficient replication capacity in human hosts. Receptor availability in human airways is a critical bottleneck for avian influenza virus transmission among humans. Influenza A virus HA initiates infection by binding glycans with a terminal sialic acid moiety on the target cell surfaces. Human influenza viruses preferentially bind to glycans terminated with α2,6-linked sialic acids (“human-type” receptors), which are abundantly expressed on the entire human airway epithelium, whereas avian influenza viruses prefer α2,3-linked sialic acids (“avian-type” receptors), which are only restricted to the lower human airways.^8^^,^^10^ Due to the scarcity of α2,3-linked sialic acids within the human upper respiratory tract, avian influenza viruses need to travel deep into the lower respiratory tracts to bind to their receptors and initiate infection. The lack of replication in the upper respiratory tract reduces viral shedding into the environment and subsequent transmission. High occurrence of severe diseases from avian influenza virus infections is attributed to their replication in the lower respiratory tracts because of skewed receptor distribution.

Although some binding to human-type receptors have been detected, bovine-associated H5N1 viruses generally seem to retain a strong preference for avian-type receptors,^33^^,^^34^^,^^39^^,^^49^^,^^50^ with expanded binding breadth.^49^ Amino acid mutations within and around the receptor binding site are shown to alter the binding specificity of HA between avian-type and human-type receptors. Studies on historical pandemic viruses have identified such key amino acid substitutions, including E190D/G225D for H1 subtype and Q226L/G228S for other subtypes.^51^ Single Q226L substitution is sufficient to switch receptor binding preference from α2,3-linked avian-type receptors to α2,6-linked human-type receptors of HA from a human isolate of bovine-associated H5N1 (A/Texas/37/2024), albeit with lower affinity than the 2009 pandemic H1 HA.^52^ Although Q226L mutation enables binding of H5 HA to α2,6-linked receptors, whether it is sufficient to confer aerosol transmission of H5N1 2.3.4.4b remains unknown. Historical HPAI H5N1 studies demonstrated that a combination of several amino acid substitutions, in addition to Q226L, is required to enable aerosol transmission in ferrets and guinea pigs.^29^^,^^30^ Further studies are warranted to understand the impact of receptor preference-switching mutations on replication fitness and transmissibility of HPAI H5N1 in animal models.

Efficient replication capacity in mammalian cells is critical in facilitating transmission, both by increasing viral shedding from donor hosts and by establishing productive infection in recipient hosts even with lower viral input. Thus, successful use of mammalian host factors for efficient replication is a key determinant for human spillover. E627K substitution in the PB2 gene is a well-defined genetic trait for mammalian adaptation.^51^ PB2-E627K promotes replication in mammalian cells by enabling support from mammalian host ANP32 proteins^53^ and increases pathogenicity in mice.^54^ Moreover, PB2-E627K contributes to aerosol transmission of HPAI H5N1 between ferrets.^30^ PB2-E627K mutation was detected in the clade 2.3.4.4b H5N1 isolate from a critically ill patient in Canada.^55^ Bovine H5N1 isolates without the PB2-E627K mutation possess the PB2-M631L mutation, which also promotes viral genome replication.^39^ The PB2 gene of swine-origin 2009 pandemic H1N1 viruses also lacks the E627K mutation but bears G590S/Q591R mutations, which promote viral polymerase activity in human cells and are structurally in close proximity to E627.^56^

Adaptive mutations in the nucleoprotein (NP) gene of human influenza viruses highlight the critical role of evading host restriction factors. Polymorphisms at residues 52 and 313 of NP determine viral sensitivity to the antiviral proteins Mx (Myxovirus resistance)^57^ and BTN3A3 (butyrophilin subfamily 3 member A3).^58^ Notably, while H7N9 avian influenza viruses have acquired resistance to both Mx and BTN3A3, H5N1 NP has not evolved these adaptations extensively. Moreover, aerosol transmission study using recombinant H5N1 reassortants with internal genes from the 2009 pandemic H1N1 virus demonstrated that PA and NS (encoding non-structural proteins) segments enable aerosol transmission.^59^ Although these segments encode multiple proteins, one possibility is that the “human-adapted” innate immune antagonists encoded in these segments (such as PA-X and NS1) enhance transmission efficiency. Characterization of adaptive mutations in NP, along with innate immune antagonists of H5N1 clade 2.3.4.4b, is crucial for understanding its transmissibility.

## Why does bovine-associated H5N1 exhibit attenuated pathogenesis in humans?

A deeper understanding of avian influenza virus pathogenesis is fundamental to assessing public health risks and guiding preparedness measures to mitigate the impact of future outbreaks.^60^ Historically, avian H5N1 influenza virus infections are associated with a >50% case fatality rate.^61^ In contrast, only one person out of 70 confirmed cases died from H5N1 infection during the current H5N1 2.3.4.4b outbreak in the United States as of May 2025.^62^ H5N1 2.3.4.4b infections are generally associated with mild clinical manifestations, such as conjunctivitis, fever, and respiratory symptoms.^63^ The basis for the relatively mild symptoms in humans infected with bovine-derived H5N1 viruses remains unclear. However, recent studies shed some light on possible mechanisms, including antagonism of host innate immune responses, cellular tropism, and the role of preexisting antiviral immunity.

Potential association of clinical manifestations and genotype has been proposed. B3.13 genotype is widely spread among dairy cattle. Notably, viral genomic sequences identified that the person who died from clade 2.3.4.4b H5N1 infection in Louisiana^62^ and a severe case in Canada^55^ were both infected with genotype D1.1, which is mainly circulating in bird species. Additional data on disease severity and side-by-side assessment of the pathogenicity of B3.13 and D.1.1 viruses in mammalian animal models should be performed to address these potential differences.

The ability to evade the host immune system is a key determinant of viral pathogenesis. NS1 is a multifunctional virulence factor with a broad capability of antagonizing the host innate immune responses.^64^ An in vitro study compared the immune evasion capacity of human and avian NS1 proteins directly by generating recombinant influenza A viruses bearing various NS1 from human or avian strains with the same backbone.^65^ Recombinant influenza A viruses bearing avian virus-derived NS1 induced less type I interferon than those bearing human NS1 proteins without affecting replication capacity in human bronchial epithelial cells. By analyzing ancestral (isolates from 1996–1997) and newer (isolates from 2016 and later) H5N1 NS1 sequences, another study identified 18 amino acid changes over the course of 20 years of evolution.^66^ The 18-amino acid substitutions increased the capability of H5N1 NS1 to inhibit host gene expressions, which is associated with decreased proinflammatory cytokine levels and attenuated virulence in a mouse model. In addition, other viral virulence factors such as PA-X and PB1-F2 may come into play by altering innate immune responses or cell death pathways. These potential mechanisms warrant further investigation.

Mild clinical manifestations with H5N1 clade 2.3.4.4b in humans may be linked to its tissue tropism and route of exposure. Conjunctivitis has been the predominant symptom in cattle H5N1 human cases,^63^ which is uncommon in historical avian influenza infections. Eyes are permissive to influenza A virus infections in mice and ferrets in vivo and human cells in vitro, with avian influenza viruses (H7 and H5) exhibiting greater ocular tropism than human-adapted strains (H1 and H3).^67–69^ While human-type receptors are present, corneal epithelial cells are rich in avian-type α2,3 sialic acid receptors.^67^^,^^69^ However, a bovine-associated H5N1 strain isolated from human eyes presenting conjunctivitis (A/Texas/37/2024) does not appear to be particularly adapted to ocular tissue, as it replicates similarly in human corneal epithelial cells to other H5N1 strains.^39^^,^^70^ Owing to the mammary gland tropism of H5N1 2.3.4.4b viruses and their excretion into cow’s milk,^19^^,^  ^24^ combined with the limited use of eye protection among infected dairy farm workers,^63^ the milking process may facilitate splash exposure of virus-laden milk to the eyes. Despite the mild ocular symptoms in humans, ocular infection with bovine-associated H5N1 has been shown to be transmissible and lethal in ferrets.^70^ Importantly, prior H1N1 infection reduced nasal viral shedding following 2.3.4.4b challenge in ocular-infected ferrets and completely blocked subsequent direct contact transmission to co-housed naïve ferrets. These findings underscore the role of cross-protective, preexisting immunity in mitigating clinical severity and transmission of bovine-associated H5N1 viruses.

## Current human immunity against H5N1 influenza viruses

The key to understanding the mild clinical manifestation and limited human-to-human transmission of 2.3.4.4b H5N1 may lie in preexisting immunity. Comparative analysis of T-cell epitopes across H5N1 and historically circulating human influenza viruses (H1N1, H2N2, and H3N2) revealed substantial conservation of both CD4 and CD8 T-cell epitopes in humans.^71^^,^^72^ Although humans are immunologically naive to the H5 HA, most individuals achieve immunity against H1 subtype in their childhood,^73^ both of which belong to antigenic group 1 and share a highly conserved stalk domain. Recent studies have provided insights into preexisting immunity against N1 resulting from seasonal and pandemic H1N1 exposures. A study reported that the low baseline NA-inhibiting antibodies against N1 from H5N1 clade 2.3.4.4b were detected in 42% of serum samples collected in 2009.^74^ In contrast, 96.8% of serum collected in 2020 contained such antibodies in higher levels. Importantly, infection with contemporary H1N1, but not with H3N2 nor immunization with seasonal vaccines, elevated antibody responses against H5N1 2.3.4.4b.^75^^,^^76^ Higher preexisting N1 immunity after the 2009 H1N1 pandemic may partially explain the differences in disease severity between the current bovine H5N1 outbreak and historical avian H5N1 outbreaks.

Animal studies show previous exposure to H1N1 or H3N2 seasonal influenza viruses gives protection against 2.3.4.4b challenge.^70^^,^^72^^,^^77–79^ Intranasal H1N1 preexposure protected ferrets and mice from lethal H5N1 2.3.4.4b infection and reduced respiratory viral titers, as well as limited systemic viral dissemination.^70^^,^^77^^,^^78^ Similarly, repeated oral administration with H1N1 virus in cow milk protected mice from oral H5N1 2.3.4.4b challenge without inducing hemagglutination inhibition titers against H5N1^,77^ suggesting neutralizing antibody-independent protective mechanisms. In agreement with this, Le Sage et al. reported that while H1N1 infection failed to elicit H5N1 2.3.4.4b neutralizing antibody responses, it did induce anti-N1 antibodies that cross-react with a recent avian N1 in serum.^78^ H3N2 preexposure was shown to induce anti-NP serum antibodies and partially protect against H5N1 2.3.4.4b challenge, suggesting potential protective roles of NP-targeting preexisting immunity.^79^ Although it is reassuring that cross-protective immunity from infection or vaccination to seasonal influenza viruses can mitigate disease from H5N1 infection to some extent, it should be noted that these studies assessed the effect of relatively recent H1N1 exposure (21 to 98 days before H5N1 challenge). Whether more remote exposure confers similar protection in humans remains unknown. Furthermore, the relative contributions of preexisting mucosal versus systemic immunity to human influenza A viruses in protecting against H5N1 remain unclear and need further investigation (Fig. 1B).

Preexisting cross-protective immunity may act as herd immunity and limit the spread of H5N1 viruses at a population level. This could partially explain why H5N1 has not resulted in efficient human-to-human transmission or a pandemic to date. However, susceptibility to H5N1 infection varies between individuals and is shaped by heterogeneity in immune status to influenza A viruses. Factors such as age, underlying health conditions, and, importantly, immune history to influenza A viruses contribute to this variability. A key component of immune history is antigenic imprinting, also known as “original antigenic sin.” This refers to the phenomenon that the immune system’s first exposure to an antigen shapes subsequent immune responses to related antigens throughout life.^80^ For instance, individuals first exposed to H3N2 tend to mount stronger immune responses to H3N2 than to H1N1, even if H1N1 infection occurs later in life. Originally described for influenza,^81^ similar imprinting effects have been observed in other viral infections, such as SARS-CoV-2.^82^

Because most individuals have been infected with influenza A viruses within their first 5 years of their lives,^73^ circulating viruses around the birth year would influence the immunity to influenza A viruses later in life. Since the 20th century, influenza A viruses have caused 4 pandemics: H1N1 in 1918 and 2009, H2N2 in 1957, and H3N2 in 1968.^60^ Following each pandemic, the pandemic viruses turned endemic in humans and predominantly circulated as seasonal viruses and disappeared upon the emergence of new pandemic strains. An exception to this is the H3N2 virus that continues to circulate to date. In 1977, an H1N1 virus reemerged and started to co-circulate with H3N2 viruses.^2^ Thus, individuals born before 1968 are likely “immunologically imprinted” to group 1 influenza A viruses (eg H1, H2, and H5 subtypes). Conversely, those born between 1968 and 1977 are likely antigenically imprinted to group 2 (eg H3 and H7) influenza A viruses. Indeed, the predominance of severe H5N1 cases was associated with estimated antigenic imprinting to group 2 influenza A viruses, while severe H7N9 prevalence followed the opposite pattern.^83^ Antibody cross-reactivity to group 1 and group 2 influenza A viruses was associated with birth year cohort, reflecting the strong influence from antigens initially exposed in childhood, even after >60 years from the encounter.^84^ A similar imprinting effect is also observed for antibodies directed to NA.^75^ Of note, older adults born before 1968 (immunologically imprinted to group 1 viruses) possess high levels of clade 2.3.4.4b H5 cross-reactive antibodies, suggesting greater resistance to H5N1 infection compared to younger individuals.^84^ Two doses of inactivated H5 vaccines elevate H5-reactive antibodies across all age groups, including those targeting the conserved stalk domain, with the most pronounced response observed in children.^84^ Given that most currently licensed H5 influenza vaccines also follow a 2-dose regimen and are inactivated-virus based,^85^ individuals with low baseline immunity would benefit from vaccination. In summary, preexisting cross-reactive antibodies to clade 2.3.4.4b H5, avian N1, and NP, along with conservation of T-cell epitopes, likely contribute to reduced disease severity and limit the spread at the population level. Introduction of H5 vaccines could further strengthen protective immunity.

## Conclusions and outlook

As described in this mini review, avian influenza H5N1 has caused a panzootic in birds and mammals and an increase in human cases during the past few years. Importantly, the first recorded human case of Influenza H5N5, a different avian influenza strain from H5N1, was detected in the state of Washington on November 13, 2025 (New reference: CDC. 2025. Weekly US Influenza Surveillance Report: Key Updates for Week 46, ending November 15, 2025. Available from: https://www.cdc.gov/fluview/surveillance/2025-week-46.html). This highlights the current potential for H5N1 and other novel strains, such as H5N5, to heighten pandemic concerns. This exemplifies the necessity to increase preparedness and countermeasures against a future influenza pandemic. Vaccination is one of the most effective strategies for preventing illness and the spread of infectious diseases, including influenza viruses. The COVID-19 pandemic demonstrated how new technologies, such as mRNA vaccines, enable rapid vaccine development and deployment, saving millions of lives and substantially decreasing the burden of an emerging pandemic. A similar employment of mRNA technology for H5N1 2.3.4.4b is under preclinical development.^86^^,^^87^ To prepare for future influenza pandemics, efforts are underway to develop broadly protective or universal influenza vaccines capable of conferring immunity against a wide range of influenza virus strains (Fig. 1C). In addition to the breadth of protection, the anatomical site of immune induction is a critical consideration. While parenteral vaccines reduce disease severity by inducing systemic immunity, they do not efficiently generate mucosal immunity at the airway mucosal surfaces. As a result, they offer limited protection against infection and onward transmission. To address this gap, it is critical to develop effective mucosal vaccines that have the enhanced potential to block transmission of influenza. Intranasal administration of adenovirus-vectored H5 influenza vaccine induced robust and durable mucosal humoral responses in humans.^88^ Additionally, unadjuvanted intranasal H5 protein boosters elicit H5-reactive systemic and mucosal antibodies in H1N1 preimmune mice.^89^

While some molecular barriers to mammalian adaptation and transmission of avian influenza viruses have been identified, a deeper understanding of these mechanisms is essential for pandemic preparedness. The development of biosafe platforms that allow safe testing of mutations with the potential to enhance viral transmission or adaptation in mammals is needed (Fig. 1A). A pseudovirus-based approach was employed to characterize mutations that improve binding of H5 HA to human receptors.^90^ Importantly, the pseudovirus-based approach is not suitable for measuring pathogenic and transmission potential, and other tools are needed to test these aspects. To address this while mitigating the risk of enhancing human transmission potential, one approach could be to use trans-complementation systems. Trans-complementation systems utilize replication-defective viruses complemented by an essential protein in *trans* to allow replication only in the desired system, thereby diminishing the risk of developing gain-of-function mutant viruses. However, their implementation in animal models has not been described and thus is a critical area of future research in pathogens with pandemic potential.

Influenza viruses can use multiple host species for evolution, reassortment, and adaptation to eventually acquire efficient human-to-human transmission capacity. Each of these hosts, such as pigs and birds, has their own evolutionary pressures to prevent or facilitate influenza virus replication and evolution. The One Health approach recognizes the interdependency of human and animal health and aims to optimize the balance between the health of humans, animals, and their ecosystems.^91^ Therefore, it is important to study the virology and immunology of relevant animal hosts in response to viruses from a One Health perspective (Fig. 1B). For instance, elucidating ecological and immunological factors of natural and intermediate hosts that facilitate replication of zoonotic strains might inform pathways to prevent influenza virus evolution, spillover, and spread. A potential research avenue is to perform CRISPR-based screens to identify proviral and antiviral genes from these animal hosts in avian influenza virus replication.^92^ Additionally, studying the magnitude and durability of immune responses (ie, antibody production and neutralization capacity) after infection^93^ is needed to inform the development of future vaccination approaches for cattle or poultry.^94^

Finally, regaining the trust of the general public is a crucial area of opportunity for scientists. Scientists in the relevant areas of research should engage with the general public and their local communities to communicate the risks of viral infections for future pandemics (Fig. 1D). Moreover, it is important to communicate research efforts underway to safeguard society against a potential avian influenza pandemic. In a collaborative effort, the continuous practice of scientific communication and transparency with the general public will become essential to prevent or respond to a future influenza pandemic.

## Data Availability

No new data were generated or analyzed in this study. Data sharing is not applicable to this article, and all information discussed is available within the cited references.
